# Topical cannabidiol is well tolerated in individuals with a history of elite physical performance and chronic lower extremity pain

**DOI:** 10.1186/s42238-023-00179-8

**Published:** 2023-03-30

**Authors:** Nicole Hall, Bradie James, Mohammad Alfrad Nobel Bhuiyan, Erin Crane, Carlie Falgout, Kevin Sean Murnane

**Affiliations:** 1 Department of Pharmacology, Toxicology & Neuroscience, Louisiana State University Health Sciences Center at Shreveport, Shreveport, LA USA; 2 Louisiana Addiction Research Center, Louisiana State University Health Sciences Center at Shreveport, Shreveport, LA USA; 3 Tiger Research Group, Dallas, TX USA; 4 Department of Medicine, Louisiana State University Health Sciences Center at Shreveport, Shreveport, LA USA; 5 Department of Psychiatry and Behavioral Medicine, Louisiana State University Health Sciences Center at Shreveport, Shreveport, LA USA

**Keywords:** Cannabidiol, Topical, Pain, Disability, Elite Athlete, Cannabinoid

## Abstract

**Introduction:**

Cannabidiol (CBD) is a potential therapeutic for pain management. Yet, there exists a dearth of studies of its tolerability and efficacy, especially in special populations. Former elite athletes are a special population both susceptible to chronic pain and also highly trained and attuned to assess medication tolerability concerns. The purpose of the present open-label pilot study was to assess the tolerability of CBD in this population.

**Materials and methods:**

Retrospective analysis was conducted in deidentified data from 20 individuals who were all previously professional athletes in US/American football, track and field, or basketball, with careers ranging from 4 to 10 years. Participants received topical CBD (10 mg twice daily by controlled dispenser) for chronic pain resulting from acute lower extremity injuries. Assessments of tolerability and secondary analyses of pain, pain-related disability, and activities of daily living were collected by self-report over the 6-week study period. Data were analyzed by descriptive statistics, pairwise *t*-test, and linear regression.

**Results:**

Seventy percent of participants completed the study. Of the individuals who completed the study, 50% reported minor adverse effects, none of which required medical attention, and 50% did not report any adverse effects. The mostly commonly reported effects were skin dryness (43% of study completers) and skin rash (21% of study completers), which rapidly resolved. There was a significant improvement in self-reported pain levels (intake mean 3.5 ± 0.29; exit mean 1.7 ± 0.23; *P* < 0.001) and pain-related disability, including family and home responsibilities, life support activities, occupational activities, recreational activities, self-care, sexual function, and social activities (all *P* < 0.001).

**Discussion:**

To the best of our knowledge, this is the first study to assess CBD treatment in elite athletes, who are disproportionally susceptible to disabling injuries. Topical administration of CBD was tolerated well by this population and resulted in only minor adverse effects. As elite athletes are trained and attuned to assess their own bodies due to their professional lives, this population is likely to detect tolerability concerns. However, this study was limited to a convenience sample and self-reported data. These pilot findings warrant further study of topical CBD in randomized and controlled studies of elite athletes.

## Introduction

The potential therapeutic use of *Cannabis* and related plants for numerous medical indications is an area of great interest and research. Although hundreds of active chemical compounds are present in *Cannabis*, the preponderance of research has focused on the cannabinoids delta-9-tetrahydrocannabinol (∆9-THC) and cannabidiol (CBD). CBD has garnered particularly strong interest as it seems to possess many of the therapeutic effects of cannabis while lacking both psychoactive effects and a potential for misuse or diversion.

The endocannabinoid system consists of the cannabinoid receptors, endogenous ligands for these receptors, and the enzymes involved in the synthesis and degradation of these endogenous ligands. The primary components of the endocannabinoid signaling system (including its receptors and endogenous ligands) are present in the synovium of both osteoarthritic (OA) and rheumatoid arthritic (RA) patients, with evidence supporting amelioration of the pathophysiology of joint pain (Richardson et al. 2008). However, the neuropharmacology of CBD has not yet been clarified, and a diversity of endocannabinoid and non-endocannabinoid mechanisms have been proposed (Mlost et al. 2020).

Consistent with this physiology, patients suffering from chronic arthritic and musculoskeletal pain represent the some of the most prevalent users of medicinal cannabis (Ware et al. 2005). The use of CBD in particular has been rapidly increasing with changes in its legal status and availability, which has only intensified with the market approval of Epidiolex® (Boyaji et al. 2020). Individuals specifically with lower extremity related osteoarthritis are prevalent users of CBD products, and most commonly use CBD as an oral tincture or in a topical application (Deckey et al. 2021). Despite the growing prevalence of using CBD for joint pain, there is scant, well-vetted research on its tolerability and efficacy in humans. CBD alone groups are rare and there are few studies of its benefits (Mlost et al. 2020), especially topical CBD. Its safety profile, lack of diversion potential, and growing prevalence of use warrant studies of the tolerability and efficacy of CBD for joint pain and other forms of chronic pain, especially in special populations and according to its most common routes of administration. Topical CBD has been studied in symptomatic peripheral neuropathy of the lower extremities (Xu et al. 2020), for thumb basal joint arthritis (Heineman et al. 2022), and following total knee arthroplasty (Haffar et al. 2022), with mixed results. Studies in unique populations such as elite athletes are even more sparse.

Due to the recent elimination of CBD from the list of prohibited substances by federations and international institutions of sports in 2018, the use of CBD among athletes is becoming more common, making this a particularly relevant population of study (Rojas-Valverde 2021). It has been proposed that CBD could improve the efficiency of recovery during and after exercise in athletes, and athletes specifically desire to use topical CBD in order to limit systemic exposure, which is perceived to further increase safety. The current study was therefore conducted to assess the tolerability of topical CBD in elite athletes. It focused on elite athletes for the reasons discussed but also because elite athletes are highly trained to observe and recognize changes in their bodies, through many years of contact with elite medical professionals as well as their common reliance on their physical health for economic wellbeing. They therefore represent a sensitive group in which to assess the tolerability of topical CBD, and evidence of tolerability in this group is highly supportive of tolerability in the general population. The study focused on athletes who had played US/American football, track and field, and basketball since the linear biomechanical movements in these sports share common features. Furthermore, athletes from all of these sports are disproportionately vulnerable to lower extremity injuries from the repeated excess force on knees, ankles, and feet required for their participation. The primary hypothesis was that topical application of CBD would be tolerable with minimal adverse effects. Secondarily, we assessed changes in pain and ease of activities of daily living in the participants throughout the 6-week study period.

## Materials and methods

### Participants

Tiger Research Group (TRG) is a private organization with the mission to optimize the safety and efficacy of cannabinoid therapy through education and research that is located in Dallas, Texas. Twenty individuals, who were all volunteers made up of former collegiate athletes who advanced to a professional league within their chosen sport, were recruited by the TRG. Participants were recruited from national sports conferences and rehabilitation facilities frequented by former professional athletes through word of mouth. No specific advertising was used for the study. The participants’ collegiate play spanned from 3–5 years in sports including what can be referred to as US football or American football (only male participants), track and field (both male and female participants), and basketball (only female participants). These former collegiate athletes also all played or participated in a professional league within their respective sport, with careers ranging from 4 to 10 years. Additional inclusion criteria were that the participants had experienced an acute injury in the lower extremities that caused chronic pain for at least 3 months before study initiation and self-reported a pain level at intake of at least 2 on the pain journal (described later).

Prior to the initiation of the research study, the athletes experienced chronic pain that had lasted a minimum of 3 months. This criterion was set because the International Classification of Diseases defines chronic pain as persistent or recurrent pain lasting longer than 3 months (Treede et al. 2015). As is routine practice with elite athletes, several treatment options were explored. Treatment modalities varied between subjects but included visits to primary care doctors, athletic trainers, chiropractors, and massage therapists for pain relief. In some individuals, surgical repair was required for the injury leading to post-surgical pain and discomfort. Use of prescription and over the counter pain relieving medications such as opioids, non-steroidal anti-inflammatory drugs (NSAIDs), and acetaminophen were also treatment options utilized before initiation of the study. Prior to the 6-week study period, all non-study treatment modalities were discontinued, including any pharmacotherapy with opioids, NSAIDs, and acetaminophen.

### Drug administration

**Fig. 1:**
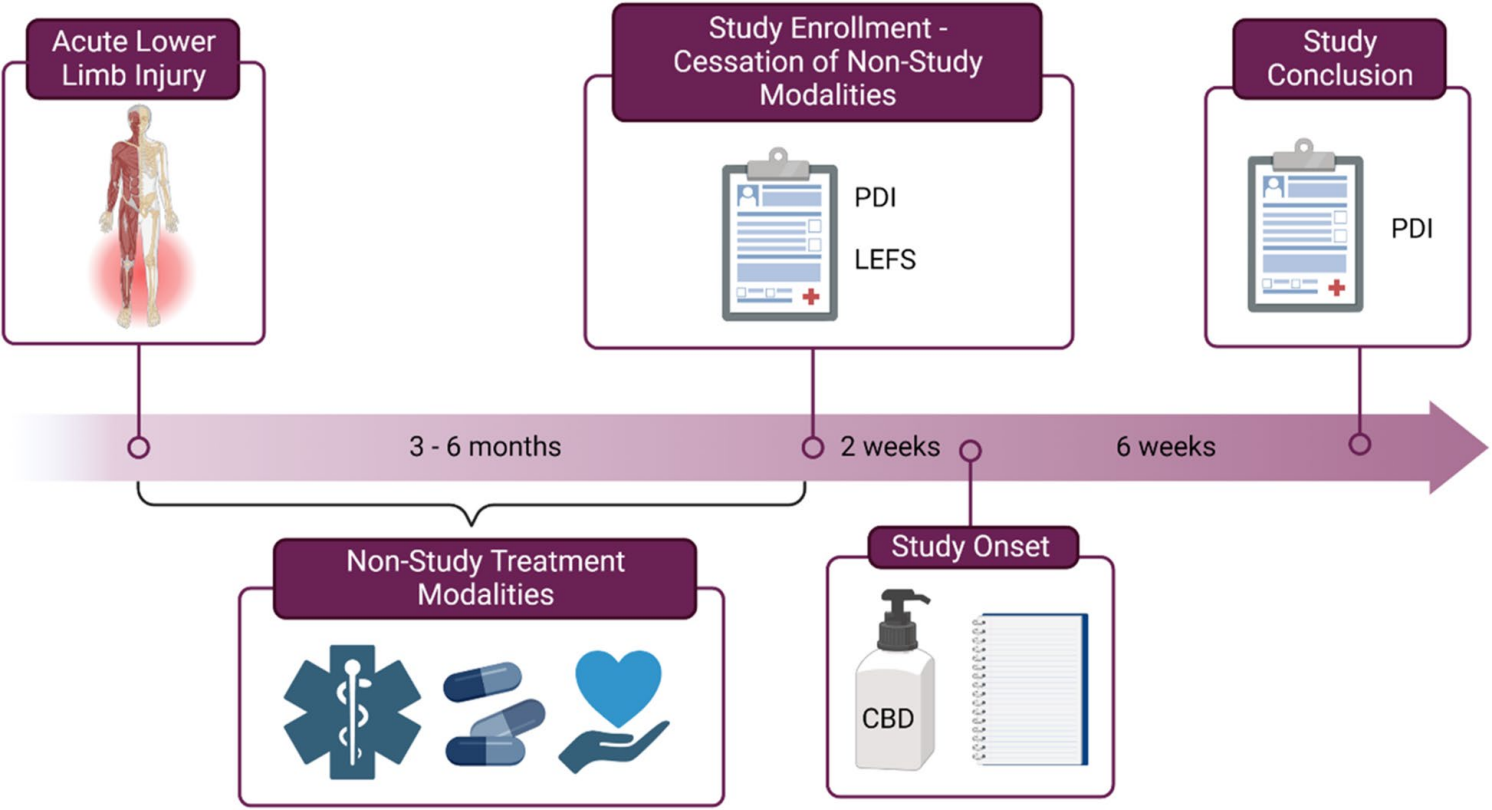
Timeline of the study design. The participants reported experiencing a lower extremity injury 3–6 months prior to enrollment. They then underwent various non-study related therapeutic modalities, which they self-reported to be ineffective. At study enrollment, they were asked to discontinue all of these modalities during the study period. At intake, participants completed the Pain Disability Index (PDI) and the Lower Extremity Functional Scale (LEFS). They also began to complete a daily pain journal. Participants received cannabidiol (CBD) treatment at 10 mg twice daily by controlled topical dispenser for six weeks. At the conclusion of the study, they completed their last entry in the pain journal as well as a second PDI

Participants were treated with a twice daily, topical formulation with 10 mg CBD as the active ingredient (Fig. 1). The proprietary CBD formulation was provided to TRG by an independent commercial source. This formulation was composed of a primary active agent of CBD. It also contained essential oils such as lemongrass, ylang ylang, and wintergreen, as well as camphor. These excipients could provide some level of chemical enhancement of skin permeation but were not specifically used to promote systemic absorption. It was applied as a cream using a controlled dispenser that delivered 5 mg/0.5 ml in each dispensing. Participants were asked to use the dispenser twice with each application and to make two applications per day. Each application was allowed to permeate the skin and was not removed by the participant. Topical administration was chosen because oral tincture and topical administration are the two most common routes of administration of CBD (Deckey et al. 2021). Topical administration typically limits systemic absorption, potentially improving the safety profile and tolerability. Topical administration is also used in the standard of care for joint pain, with several branded formulations of NSAIDs, capsaicin, and other active agents on market. Athletes typically have a history of use of topical analgesia and a preference for topical administration because of its perceived safety. Topical agents often reach the joint or nerve endings but do not appreciably provide systemic delivery unless they have specific physiochemical properties or undergo permeation enhancement with chemicals or other techniques. A necessary property for passive systemic delivery is moderate lipophilicity (log P of 1–3), which is often not the case with phytocannabinoids as they are highly lipophilic (Tijani et al. 2021). In this regard, CBD is a highly lipophilic molecule (log P 5.8) that produces limited skin permeation even with chemical enhancement by essential oils (Junaid et al. 2022). However, its high lipophilicity increases the probability that it will accumulate or form depots that can lead to local skin irritation, including skin erythema and xerosis. The CBD treatment (10 mg, twice daily) was administered by a controlled dispenser provided to the participants. The dispenser contained 300 mg of CBD and provided controlled applications of 5 mg/0.5 ml. Each participant was provided with 4 dispensers at the initiation of the study. The 20 mg daily dose was chosen because it is in line with available doses, the typical dose range of topical analgesia, and recent clinical studies of topical CBD for pain. However, we note that very limited information is available on the appropriate doses range for topical CBD and that this is likely a conservative dose given the higher doses of oral CBD used for psychiatric indications such as anxiety and the approved dose range of Epidiolex®.

### Assessments

At intake, participants completed several instruments to gauge pain, disability, daily function, and perceived responses to treatment. The Pain Disability Index (PDI) was used to evaluate the degree to which the participant’s pain impacted daily life. This instrument has been used to evaluate the perceived disability caused by pain and to determine the efficacy of rehabilitation and/or treatment on pain related function (Grönblad et al. 1993; Gross et al. 2012; Pollard 1984; Tait et al. 1990). The PDI was evaluated on a Likert scale (10 = worst disability; 0 = no disability). Subjects were given the form at intake and exit to assess whether self-report measures of pain-related impairments in the ability to complete tasks in specific functional domains changed over the course of the 6 weeks of CBD administration. The domains examined included family or home responsibilities, recreation, social activities, occupational activities, sexual function, self-care, and life-support activities. The difference in pre- and post-treatment scores were calculated by subtracting the intake score from the exit score of each subject. The mean and standard deviation of each domain were calculated both pre- and post-treatment.

The Lower Extremity Functional Scale (LEFS) is a self-reported questionnaire that was used to assess functional disability as indexed by difficulty with activities of daily living. This instrument has been used to assess a participant’s initial function as well as ongoing response to treatment for lower extremity pain (Binkley et al. 1999; Lieberz et al. 2022; Mehta et al. 2016). The LEFS has been reported to have strong construct validity and test–retest reliability (Binkley et al. 1999) and has been reported to be appropriate for use in individuals with lower extremity musculoskeletal dysfunction and pain (Binkley et al. 1999; Fukuda et al. 2010; Leibbrandt and Louw 2018). The scale is composed of 20 items that are evaluated on a Likert scale (0 = extreme difficulty; 4 = no difficulty) across many activities of daily living. The maximum possible score is 80 points, and higher scores indicate better function. Individuals were given this survey on intake to assess the domains with which the subjects had the most difficulty. The activities of daily living examined were conducting usual work activities, conducting usual hobbies or recreational activities, getting into or out of a bath, walking between rooms, putting on shoes/socks, squatting, lifting, performing light activities, performing heavy activities, entering or exiting a car, walking 2 blocks, walking a mile, going up or down stairs, standing for 1 h, sitting for 1 h, running on even ground, running on uneven ground, taking sharp turns while running, hopping, and rolling over in bed. The mean and standard deviation of participant’s scores were calculated for each domain. The tasks were ranked in order from easiest to most difficult.

The individuals completed a pain journal over the course of treatment. Subjects were asked to self-assess their pain each morning (0 = no pain; 5 = worst pain), as well as their symptom improvement for the day after taking their treatment (1 = no improvement; 5 = complete improvement in symptoms/complete disappearance of symptoms). This was done to assess whether overall mean pain levels decreased over course of treatment (from week 1 to week 6) and whether worse baseline pain levels were associated with greater pain relief. The average pain level and average symptom relief of each participant for each week was calculated.

### Data analysis

This deidentified and retrospective data analysis was approved by the Institutional Review Board of Louisiana State University Health Sciences Center – Shreveport (LSUHS). Following IRB approval, TRG provided entirely deidentified data from these assessments to researchers at LSUHS. All private health information was removed from these participant records. The score from the intake pain disability index was subtracted from the exit score to calculate a mean difference. The across subject average of each task on the lower extremity functional scale was calculated and the tasks were ranked in order of difficulty. The mean pain and symptom relief levels of each participant for each week recorded were calculated from the pain journals. An overall average per week was calculated as a measure of improvement over time. Descriptive statistics were used to assess baseline characteristics. Numerical data are shown as mean ± the standard error of the mean (SEM). All data were tested for normality using the Shapiro–Wilk test. Pairwise *t*-test was used to evaluate mean differences before and after treatment, and post hoc comparisons were performed by Bonferroni posttest. Pearson correlation was used to check the correlation between pain level and symptom relief level by week of treatment. Moreover, a linear regression adjusted by week was also used to evaluate the relationship between pain level and symptom relief over time. As a sensitivity analysis, the non-parametric Wilcoxon signed ranks test was utilized and found similar results. A value of *P* < 0.05 was considered statistically significant. All statistical analysis were performed using R (Version 4.2.1).

## Results

Twenty volunteers, all of whom experienced an acute injury to the lower extremities which caused chronic pain lasting between 3 and 6 months, were recruited into the study. The participants were treated over a period of 6 weeks with a twice daily, proprietary, topical formulation with 10 mg CBD as the active ingredient. The cohort had a mean age of 28.7 ± 1.5 (range 26–31) years and included 14 males (70%) and 6 females (30%). Demographic information is included in Table 1. Fourteen volunteers (70%) completed the study. Six dropped by day four for unspecified reasons and were not included in the statistical analysis of the assessments. Two participants failed to self-report on four or less of the tasks assessed in the LEFS. One participant did not complete the pain level and symptom relief journal past week three.

**Table 1:** Demographics and educational attainment of the study participants. Data are subdivided by the age, gender, race, and highest education completed of the participants. These data are presented for all 20 participants who enrolled in the study. The *n* represents the raw number of subjects. The percentage was calculated from all 20 participants

	**Categories**	***n***	**%**
**Age (years)**	20–29	13	65%
	30–39	7	35%
**Gender**	Male	14	70%
	Female	6	30%
**Race**	European American	4	20%
	African American	15	75%
	Asian American	0	0%
	Latino/Latina	1	5%
**Highest education completed**	High school diploma/GED	1	5%
	Some college	8	40%
	Undergraduate degree	3	15%
	Some graduate school	3	15%
	Masters degree	5	25%

A total of seven subjects (50% of those who completed the study) reported adverse events (Table 2) possibly related to the CBD treatment, although none of the reported side effects interfered with the participant’s ability to complete the study nor did they require hospitalization or other medical care before resolving without intervention. All of the women who completed the study (28.6% of total participants completing the study) described skin conditions including skin rash and dryness. Three female participants experienced a rash, although none of the male participants relayed the same. The rash began on day 5 of treatment at which time subjects were instructed to continue use of the formulation on another location of the lower extremity. By day 7 of treatment, the rash was gone, and no subsequent rashes were reported in any treatment location. All female participants and two male participants (42.9% of total participants completing the study) felt skin dryness in the treatment area beginning on day 1 or 2 and lasting no more than a day. Cold sweats were reported by one male participant, and heart racing was reported by single different male participant.

**Table 2:** Complete list of all reported adverse events in the study. Data are subdivided by the type of adverse effect. The adverse effects are ranked from most to least commonly reported. These data are presented from the 14 participants who completed the study. The *n* represents the raw number of subjects. The percentage was calculated from the 14 participants

Adverse events	Female (n)	Male (n)	All patients (n, % of participants)
Skin dryness	4	2	6, 43%
Rash	3	0	3, 21%
Cold sweats	0	1	1, 7%
Tachycardia	0	1	1, 7%

**Fig. 2:**
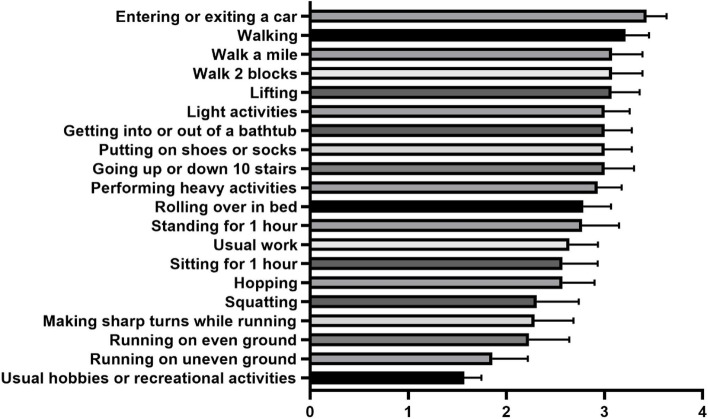
Activities of daily living assessed on the Lower Extremity Functional Scale. Data are subdivided by the type of activity. The activities are ranked from least to most difficult. These data are presented from the 14 participants who completed the study. Data are presented as the mean value and errors bar represent the standard error of the mean (SEM)

The 20 activities evaluated in the LEFS at study intake were compared by pairwise *t*-test with Bonferroni correction to determine which tasks presented the participants with the most difficulty. The tasks were ranked by order of difficulty from easiest (mean = 3.4 ± 0.202) to hardest (mean = 1.6 ± 0.173) (Fig. 2). The tasks that the participants experienced the most functional disability with at study onset were running on uneven ground (mean = 1.9 ± 0.361), participating in usual hobbies and/or recreational activities (mean = 1.6 ± 0.173), and making sharp turns while running (mean = 2.3 ± 0.398).

The difference in scores from beginning to end of study revealed that 100% of subjects reported improvement in disability related to pain in the domains of family/home responsibilities and sexual function; 93% of subjects reported improvement in recreation, social activities, occupational activities, and life-support activities; and 86% reported improvement in self-care. The pre- and post-treatment means of the seven functional domains assessed by the PDI were compared using pairwise *t*-test with Bonferroni correction. There was a significant decrease in the reported disability due to pain of the participants at exit compared to intake (Table 3) indicating the treatment provided the participants a better quality of life.

**Table 3:** Mean differences between intake and exit scores on the Pain Disability Index (PDI). Data are subdivided by the type of activity. The activities are ranked according to self-reported disability at intake, from highest to lowest level of self-reported disability. These data are presented from the 14 participants who completed the study. Data are presented as the mean value and the standard error of the mean (SEM). Significance was determined using the pairwise *t*-test. ***p < 0.001

**Activities**	**PDI intake (mean ± SEM)**	**PDI exit (mean ± SEM)**	***P* value (p.adj.)**
Sexual function	7.57 ± 1.99	0.143 ± 0.363	***
Occupational activities	7.29 ± 2.55	2.36 ± 0.842	***
Recreational activities	7.14 ± 1.99	3.07 ± 0.997	***
Life support activities	6 ± 2.48	1.57 ± 1.22	***
Family/home responsibilities	6 ± 1.75	2.79 ± 1.12	***
Social activities	6 ± 2.22	2.5 ± 0.760	***
Self-care	5.43 ± 2.31	1.93 ± 1.44	***

**Fig. 3:**
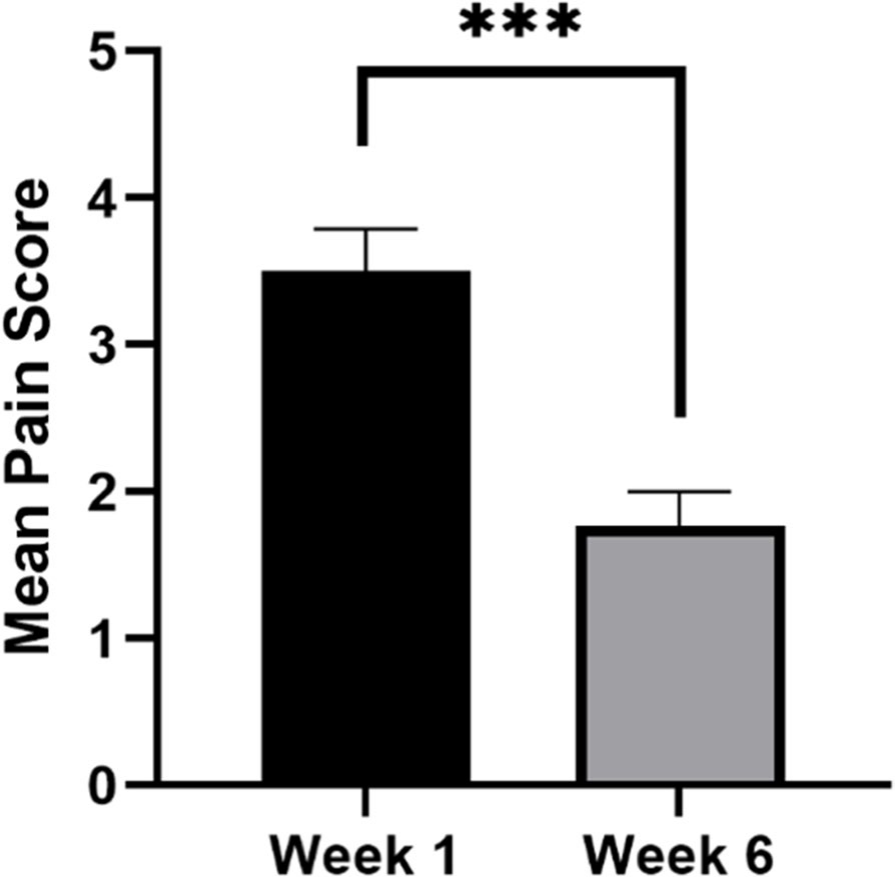
Change in pain level from intake to exit as reported on a self-report pain journal. Subjects were asked to rank their pain each morning on a scale from 0 is equal to no pain and 5 is equal to the worst possible pain. Ordinate: Mean score on the pain journal. Abscissa: scores at week 1 versus scores at week 6. Significance was determined using the pairwise *t*-test. ***p < 0.001. Error bars represent the standard error of the mean (SEM)

**Fig. 4:**
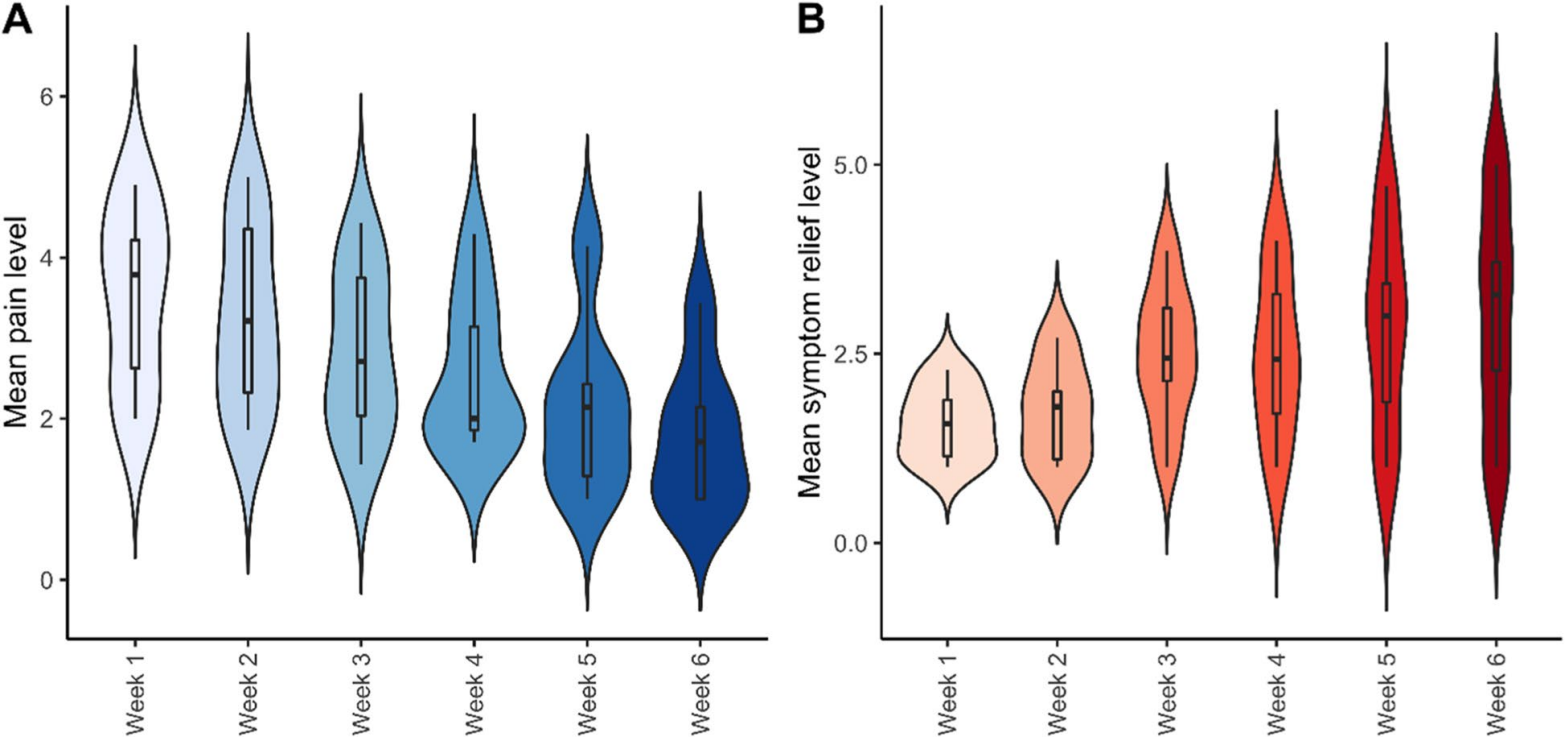
Changes in pain level and symptom relief over the course of the cannabidiol treatment. Change in pain level and symptom relief as reported on a self-report pain journal. Subjects were asked to rank their pain each morning on a scale where 0 is equal to no pain and 5 is equal to the worst possible pain. They were also asked to record their symptom improvement on a scale where 1 is equal to no improvement and 5 is equal to complete improvement. Ordinate in **A**: mean score on the pain journal. Ordinate in **B**: mean symptom improvement as recorded in the pain journal. Abscissa for both: scores from week 1 to week 6. Error bars represent the standard error of the mean (SEM)

Comparing the mean pain level from the start of treatment at week 1 to the completion of the study period at week 6 found that participant’s journal scores displayed significant improvement over the course of the study (Fig. 3). A Pearson correlation revealed a significant overall correlation between pain level and symptom improvement over the course of the treatment (*R* = 0.42, *p* = 0.00011) with the mean pain level decreasing at the same time the symptom improvement increased (Fig. 4).

## Discussion

This study evaluated the tolerability of a topical CBD treatment in a cohort of former elite athletes experiencing chronic lower extremity pain. Of the twenty recruited volunteers, 70% tolerated the treatment to study completion, and 30% reported minor adverse effects which did not affect their ability to complete treatment. It was determined that all subjects experienced significant improvements in both quality of life, as assessed by the PDI recorded at intake and exit, and relief of pain, as measured by the decrease in pain level over time recorded in the pain journals.

There are few studies examining safety and tolerability of CBD alone for treatment of pain or disease, especially topical CBD. Topical CBD has been studied in symptomatic peripheral neuropathy of the lower extremities (Xu et al. 2020), for thumb basal joint arthritis (Heineman et al. 2022), and following total knee arthroplasty (Haffar et al. 2022). Using a topical CBD oil in a randomized, double blind, placebo-controlled study of patients with symptomatic peripheral neuropathy of the lower extremities, Xu et al. reported statistically significant reductions in pain domains assessed by the Neuropathic Pain Scale including intense (*p* = 0.009), sharp (*p* < 0.001), itchy (*p* = 0.001), and cold (*p* < 0.05) when compared to placebo controls (Xu et al. 2020). However, across these studies, there were mixed results on its capacity to control the symptoms of these various indications. Nevertheless, topical CBD was well tolerated within these populations with minimal adverse effects. Nimalan et al. studied the use of cannabis-based medicinal products, which included both CBD and THC, in a case series of cancer-related palliative care patients and, similar to the current study, determined them to be tolerated with few mild (12.5%) or moderate (6.25%) adverse events reported as lethargy, ataxia, and dysgeusia each affecting 6.25% of participants and resolving spontaneously (Nimalan et al. 2022).

To the best of our knowledge, this is the first study testing the efficacy of CBD treatment on elite athletes. The need for further research in this population is highlighted in the review by Rojas-Valverede (Rojas-Valverde 2021). The LEFS and PDI employed here are widely used and validated assessments of function and disability, although the use of self-report data can be biased by an individual’s perception. Employing a repeated measures design, as was done with the PDI, reduces the between subject variability and may help overcome this bias. The current findings support the continued development of topical CBD with all participants reporting relief from pain and almost all participants seeing a decrease in disability related to pain during the 6-week study period. However, this study has limits in that it is an open-label pilot study with an associated group size. Larger randomized, controlled, and prospective studies of topical CBD in elite athletes are necessary to conclude that CBD controls pain in this special population. Moreover, this study could have benefitted from the use of a validated pain scale, such as the McGill Pain Questionnaire or short form MPQ, administered at intake and exit (Melzack 1975).

While there has been a paucity of data on the effects of CBD as a monotherapy in pain management, the initial results reported here are suggestive of a therapeutic response. It is possible that this could be further enhanced through the combined use of CBD with other cannabinoids. Previous research has examined nabiximols, a combined product of THC/CBD in a 1:1 ratio, which has shown promising results in symptomatic pain management in multiple clinical trials (Boyaji et al. 2020). A previous clinical study described the therapeutic efficacy of CBD and Δ9-THC co-administration, in doses of 2.5 mg CBD and 2.7 mg Δ9-THC in an oral mucosa spray. After treatment sessions ranging from one to several weeks, patients reported improved sleep, reduced pain, and reduced fatigue (Serpell et al. 2014).

CBD also has shown positive findings in combination with other cannabinoids in human studies of non-joint pain. A recent study examined 177 patients with chronic cancer pain, who experienced inadequate analgesia despite chronic opioid dosing, and entered a 2-week, multicenter, double-blind, randomized, placebo-controlled, parallel-group trial. Based on changes from baseline in mean pain Numerical Rating Scale scores, a combination of CBD with THC showed significant benefits compared with placebo. While there was no CBD alone group examined, the THC alone group showed no similar benefits, suggesting that the combination was due to CBD treatment. No significant group differences were found in sleep quality and a possible worsening of nausea scores following treatment with THC to CBD compared with placebo (Johnson et al. 2010). A recent large meta-analysis found that patients with chronic neuropathic pain taking THC/CBD were 1.756 times more likely to achieve a 30% reduction in pain compared to placebo (Sainsbury et al. 2021). However, when looking at CBD alone compared to placebo, they did not find a statistical difference in this pain state (Sainsbury et al. 2021).

The preliminary therapeutic response reported in this study of topical CBD monotherapy could potentially be enhanced through techniques to increase systemic delivery. Skin delivery studies have shown steady-state plasma concentration of CBD in guinea pigs after transdermal gel application was 6.3 ± 2.1 ng/mL, which was attained at 15.5 ± 11.7 h. It is possible that systemic delivery and the clinical response to topical CBD could be further enhanced through techniques to increase systemic delivery or brain targeting, including chemical enhancement (Puri et al. 2017), active techniques such as iontophoresis or laser ablation (Bhattaccharjee et al. 2020), complex formulations (Ganti et al. 2018), or nanoformulation (Zaman et al. 2018). For example, steady-state concentrations of CBD were increased 3.7-fold in the presence of a Food and Drug Administration (FDA)-compliant skin delivery enhancer (Paudel et al. 2010). In an in vitro human skin transdermal flux model, which is the best available approximation for skin delivery in intact human subjects, the skin permeability of CBD was better than other cannabinoids and ethanol concentrations of 30 to 33% significantly increased the transdermal flux of CBD (Stinchcomb et al. 2004). There are a number of ways that range from FDA-compliant passive enhancers to more advanced active drug formulation techniques to further enhance the skin delivery of CBD. In one such study, conducted in mice, a transdermal delivery system for CBD was designed using ethosomal carriers. In vivo application of the ethosomal CBD produced a significant accumulation of the drug in the skin and in the underlying muscle and prevented the inflammation and edema induced by sub-plantar injection of carrageenan (Lodzki et al. 2003).

The exact mechanism of action of cannabidiol is unknown and has been controversial (Richardson et al. 2008). However, it seems clear that it does not appear to involve direct effects at cannabinoid receptors as it has no appreciable affinity for these receptors (McHugh et al. 2008). It is possible that it may have indirect effects on these receptors as CBD appears to exert anti-inflammatory activity by increasing concentrations of the endocannabinoid anandamide, which can then stimulate receptors. Likewise, CBD has been associated with activity at a broad range of non-CB receptors, including serotonin 1A receptor (5-HT1A), TRPV1, and PPARγ receptors that have known roles in pain and inflammation. The clearest evidence from preclinical studies is that CBD produces anti-inflammatory effects that may mitigate the painful symptoms of joint damage, which mimic the inflammatory insults of arthritic disease (Burstein and Zurier 2009). Given the promising findings reported in this study, the mechanism by which CBD controls joint pain should be pursued in future studies.

The information available in a retrospective chart review is limited to what was collected. Nevertheless, a group of elite athletes suffering from lower extremity pain reported that topical CBD is tolerable in their treatment. Elite athletes, especially those that reach professional status, as was the case for the participants in the current study, undergo many years of training in diet, wellness, self-monitoring, high level medical care, and rigorous assessments. They have substantial specialized experience in self-monitoring that was tied to their economic security and professional success. They are highly attuned to assess medication tolerability due to their professional lives. As such, this population is not only susceptible to pain but are potentially more likely than the general population to detect tolerability concerns due to their experience and training. These data therefore speak well to the safety and tolerability of topical CBD in the general population.

There are several limitations of the present study that warrant caution in the interpretation of the reported findings. The study population was a small convenience sample that likely limits its external validity. While it is likely that elite athletes represent a sensitive group in which to assess the tolerability of topical CBD, it is possible that their high health status, previous history of physical stress, or other factors could influence their perceptions of tolerability and limit the generalizability of reports in this group to the general population. All of the data collected in this study were determined by self-report rather than more objective measures or biomarkers. Likewise, there was no direct monitoring of the topical CBD administration other than by the participants. These limitations should be addressed in future research studies that build upon the study results reported here.

## Conclusions

To the best of our knowledge, this is the first study to assess CBD treatment in elite athletes. Topical administration of CBD was tolerated well by this population and resulted in only minor adverse effects. As elite athletes are trained and attuned to assess safety concerns due to their professional lives, this population is likely to detect safety or tolerability concerns. Six weeks of treatment provided significant decreases in pain and improvements in function. The data collected in a pilot population warrants further study of topical CBD in prospective, randomized and controlled studies in elite athletes.

## Data Availability

Original data will be made available upon request.
